# Spatial, temporal and genetic dynamics of highly pathogenic avian influenza A (H5N1) virus in China

**DOI:** 10.1186/s12879-015-0770-x

**Published:** 2015-02-13

**Authors:** Huaiyu Tian, Yujun Cui, Lu Dong, Sen Zhou, Xiaowen Li, Shanqian Huang, Ruifu Yang, Bing Xu

**Affiliations:** State Key Laboratory of Remote Sensing Science, College of Global Change and Earth System Science, Beijing Normal University, Beijing, 100875 China; State Key Laboratory of Pathogen and Biosecurity, Beijing Institute of Microbiology and Epidemiology, Beijing, 100071 China; Ministry of Education Key Laboratory for Biodiversity and Ecological Engineering, College of Life Sciences, Beijing Normal University, Beijing, 100875 China; Ministry of Education Key Laboratory for Earth System Modelling, Center for Earth System Science, Tsinghua University, Beijing, 100084 China; Department of Geography, University of Utah, Salt Lake City, UT 84112 USA

## Abstract

**Background:**

The spatial spread of H5N1 avian influenza, significant ongoing mutations, and long-term persistence of the virus in some geographic regions has had an enormous impact on the poultry industry and presents a serious threat to human health.

**Methods:**

We applied phylogenetic analysis, geospatial techniques, and time series models to investigate the spatiotemporal pattern of H5N1 outbreaks in China and the effect of vaccination on virus evolution.

**Results:**

Results showed obvious spatial and temporal clusters of H5N1 outbreaks on different scales, which may have been associated with poultry and wild-bird transmission modes of H5N1 viruses. Lead–lag relationships were found among poultry and wild-bird outbreaks and human cases. Human cases were preceded by poultry outbreaks, and wild-bird outbreaks were led by human cases. Each clade has gained its own unique spatiotemporal and genetic dominance. Genetic diversity of the H5N1 virus decreased significantly between 1996 and 2011; presumably under strong selective pressure of vaccination. Mean evolutionary rates of H5N1 virus increased after vaccination was adopted in China. A clear signature of positively selected sites in the clade 2.3.2 virus was discovered and this may have resulted in the emergence of clade 2.3.2.1.

**Conclusions:**

Our study revealed two different transmission modes of H5N1 viruses in China, and indicated a significant role of poultry in virus dissemination. Furthermore, selective pressure posed by vaccination was found in virus evolution in the country.

**Electronic supplementary material:**

The online version of this article (doi:10.1186/s12879-015-0770-x) contains supplementary material, which is available to authorized users.

## Background

A particular subtype of influenza A virus, highly pathogenic avian influenza (HPAI) virus H5N1, is transmitted by contact with infected birds [1]. It is epizootic in many bird populations, especially in Southeast Asia. Clade 2.2 of the virus has spread globally, including Europe, the Middle East and Africa after first appearing in Asia in 2005 [2]. The spatial spread of H5N1 avian influenza and long-term persistence of the virus in some regions has had an enormous impact on the poultry industry and presents a serious threat to the health of humans and migratory birds [3,4]. It has been 17 years since the first case in geese of H5N1 avian influenza was discovered in Hong Kong in 1996 [5]. As of 19 May 2013, H5N1 has caused 628 human cases of influenza in 15 different countries, with 374 deaths.

The high lethality and virulence of H5N1, its epizootic presence, its increasingly large host reservoir, its significant ongoing mutations, and its potential transmissibility between humans, make it one of the greatest current pandemic threats [6]. Substantial progress has been made in researching various aspects of the virus and preparing for a potential influenza pandemic [7,8]. Several studies on the global spread of avian flu using phylogenetic relationships of virus isolates have indicated that migratory bird movements, and trade in poultry and wild birds could explain the pathway for introduction events into different countries [9,10]. However, the underlying mechanism of the long-term persistence of the virus and its various spatiotemporal transmission pathways, with their corresponding genetic footprints, remain poorly understood.

China is one of the world primary producers of poultry products [11], and is among the regions most affected by H5N1 [12]. Poultry production generates 16 million tons of meat and 27 million tons of eggs annually in China [11]. In 2004, the total loss caused by HPAI virus H5N1 was 4.5 billion US dollars in China. To control H5N1 infection in poultry, many countries have implemented a vaccination policy, including China, Vietnam, Indonesia and Egypt. In Mainland China, a poultry vaccine was first used at the end of 2004. Over 55 billion doses of vaccines were applied to control the outbreaks between 2004 and 2008 [13]. Antigenic variants of H5N1 avian influenza virus have occurred along with its spatiotemporal transmission. Furthermore, vaccination may change the evolutionary dynamics of H5N1 virus [14,15]. Vaccine strains can be selected from the seeding region of Southeast Asia where its genetic and antigenic characteristics can be determined earlier for human or avian influenza [2,16]. However, the effect of vaccination in China has rarely been studied and is discussed in the present study.

Here, we applied phylogenetic analysis, geospatial techniques, and time series models to investigate the spatiotemporal pattern of H5N1 outbreaks in China and the effect of vaccination on the dynamics of virus evolution. These data, combined with spatiotemporal information on the H5N1 outbreaks and viruses, were used to understand viral evolutionary dynamics from the beginning to its circulation in China. This study aimed to illustrate the spatiotemporal pattern of H5N1 outbreaks in China, understand the role of migratory birds and poultry in contributing such a pattern, and the effect of vaccination on the dynamics of virus evolution.

## Methods

### Virus sequences and outbreak data

**Figure 1:**
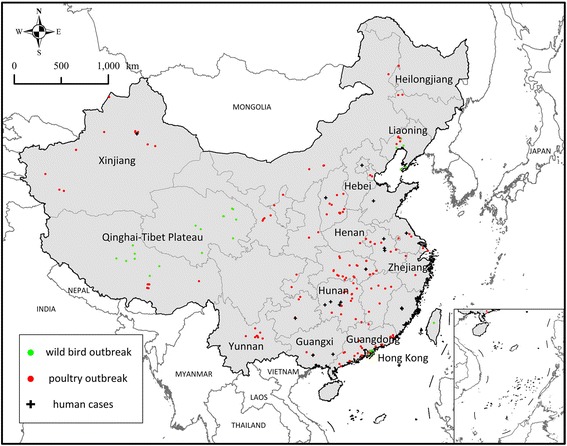
**H5N1 virus outbreaks, avian and human cases.** Outbreak records in China began in January 2004, and outbreaks were mainly concentrated in four periods: early 2004, late 2005 to early 2006, late 2007 to early 2008, and early 2009.

Full-length hemagglutinin (HA) sequences of HPAI virus H5N1 were obtained from the GenBank database (http://www.flu.lanl.gov) hosted by the National Center for Biotechnology Information (NCBI). A total of 674 non-repetitive sequences were used in the phylogenetic and selection analysis at provincial level, from 1996 to 2011 (Additional file 1: Table S1). Hosts were classified into different groups, according to the list of species affected by H5N1 avian influenza from the US Geological Survey National Wildlife Health Center. We also collected H5N1 outbreak data in China from the World Organisation for Animal Health (OIE, http://www.oie.int/) and the United Nations Food and Agriculture Organization (FAO, http://www.fao.org/home/en/), from January 2004 to December 2011. We removed redundant records and geocoded them according to their spatial information. Mainland China had 323 outbreaks in total, of which 217 were in poultry, 85 in wild birds, and 21 in humans (Figure 1). The HA sequences of HPAI H5N1 are accurate to the year, while outbreak records are accurate to the day. The HA sequences and outbreak records were not linked, and they were collected from two separate data sets.

### Spatial point pattern analysis

A point pattern analysis of H5N1 outbreaks was used to determine where the cases were spread, as well as to determine which spatial scales were optimal for disease clustering [10,17]. Choosing the right scale was critical for subsequent analyses. We used exploratory spatial statistical techniques to examine the patterns of outbreaks. Ripley’s K function describes how the expected value of a point process changes over different spatial and temporal lags [18,19]. A peak value in Ripley’s K function indicates a clustering at the scale of the corresponding lag. An estimate of spatial and temporal K function can be calculated by [20]:

1 $$ K(d)=\frac{R}{n^2}{\displaystyle \sum_{i=1}^n{\displaystyle \sum_{j\ne i}\frac{I_d\left(i,j\right)}{W_{ij}}}} $$

2 $$ K(t)=\frac{T}{n^2}{\displaystyle \sum_{i=1}^n{\displaystyle \sum_{j\ne i}{I}_t\left(i,j\right)}} $$

where *R* is the total area of the study, *n* is the number of observed events, *I*_*d*_(*i*, *j*) is an indicator function that takes a value of 1 when the spherical distance between points *i* and *j* is less than *d. W*_*ij*_ is the adjustment factor of the edge effect. In clustering, *K(d)* would be greater than *A*(*d*), which is the area of the spherical circle of arc-radius *d*; and less than *A*(*d*) under regularity. We then apply a transformation to *K(d)* to have $L(d)=R* \arccos \left(1-\frac{K(d)}{2\pi {R}^2}\right)-d$, and to plot *L(d)* against *d*. Deviation, *L*(*d*), above the zero line suggest clustering (more events within distance *d* than expected), while deviations below zero suggest regularity (fewer events within distance *d* than expected) [21]. *T* is the time span from the earliest to latest outbreak in the dataset; we changed Gregorian date to Julian date. *I*_*t*_ (*i*, *j*) is an indicator function that takes a value of 1 when the time interval between points *i* and *j* is less than *t*. All the calculations used the same study area – the extent of Mainland China.

The upper and lower bounds were determined by undertaking Monte Carlo simulations 999 times. For each simulation, we generated the same number of random points as the cases, and then calculated their K functions. To each lag, the upper and lower bounds were the minimum and maximum K values among the set of 999 simulations.

### Phylogenetic analysis

Sequences were aligned using the ClustalW algorithm [22] implemented in BioEdit (version 7.0) [23]. The optimal nucleotide substitution model was selected using the Akaike Information Criterion (AIC) [24] and a hierarchical likelihood ratio test in ModelTest [25]. Phylogenetic relationships of the 674 H5N1 HA sequences were constructed using the neighbor-joining approach and the GTR + I + Γ_4_ model of nucleotide substitution by PAUP 4.0 [26]. Tree topology reliability was tested with 1000 bootstrap replicates. The tree was rooted by A/goose/Guangdong/1/96 and structured according to the World Health Organization system of avian influenza cladistics (http://www.who.int/influenza/gisrs_laboratory/h5n1_nomenclature/en/).

Rates of nucleotide substitution per site and year were estimated by the BEAST program [27], which uses a Bayesian Markov chain Monte Carlo approach. For each analysis the constant size, exponential growth, Bayesian skyride and Bayesian skyline coalescent prior were used with the codon-based SRD06 nucleotide substitution model [28]. The strict and uncorrelated lognormal (UCLN) relaxed molecular clocks were compared by analyzing values of the coefficient of variation in the tracer. The statistical uncertainty in all analyses was reflected by the 95% highest probability density (HPD) values for parameter estimate. Additionally, the chain lengths in each analysis were run for sufficient time to achieve coverage (up to 10 million generations). To estimate the variations of evolutionary rates resulting from vaccination, the HA sequences were categorized by sampling time. We inferred the evolutionary rates in a window of 2 years with a step of 1 year forward.

To visualize the evolutionary process of the virus, we calculated the genetic distances between any pair of sequences and reprojected the distances to a genetic map using multidimensional scaling (MDS) [29,30]. MDS created a matrix of inter-point distances. These points could be reconstructed using Euclidean distance to replace genetic distance. MDS minimized the loss function as follows:

3 $$ L= \min {\displaystyle \sum_{i,j=1}^{i,j=M,N}{\left({D}_{ij}-{\delta}_{ij}\right)}^2,} $$

where *D*_*ij*_ is the genetic distance from strain *i* to *j*, and *δ*_*ij*_ is the estimated Euclidean distance. The MDS method was conducted in Matlab (version R2009b) (MathWorks, Natick, MA, USA).

### Detection of positively-selected sites

The selective pressure can be detected by measuring nonsynonymous–synonymous rate ratio (dN/dS) [31]. A ratio >1 overall represents positive selection of genes [32]. To illustrate the variation of raw mutation rate over sites, we compared the virus with the HA genes of reassortant avian influenza virus vaccine Re-1, Re-4 and Re-5 by the LPB93 method [33], with introduction of a γ distribution using the KaKs Calculator (version 2.0) [34]. We aligned the pair of sequences in a window of 57 bp with a step of 3 bp forward. Fisher’s exact test was applied for justification of small sample size. The dN/dS ratio among codons was calculated using the maximum likelihood method with the codeml program in PAML 4.5 [35,36], as well as the fixed effect likelihood (FEL) method implemented in HyPhy (version 0.99b) [37]. Positive selection was tested using a likelihood ratio test (LRT) comparing a null model (M7) that did not allow dN/dS >1 with an alternative model (M8) that did. M8 had two more parameters than M7, so that ${\chi}_2^2$ was used to conduct the LRT [38].

### Time series analysis

A cross-correlation function was used for identification of the relationship between outbreaks in different hosts. We obtained cross-correlation coefficient time series based on poultry and wild-bird outbreaks and human cases. The cross-correlation coefficient time series was calculated as:

4 $$ {\rho}_{xy}\left(\mathrm{k}\right)=\frac{\gamma_{xy}\left(\mathrm{k}\right)}{\sigma_x{\sigma}_y}\kern2em k = 0, \pm 1, \pm 2, $$

where *ρ*_*xy*_ (k) is the cross-correlation coefficient at lag k; *σ*_*x*_ and *σ*_*y*_ are standard deviations of X and Y, respectively; and *γ* is the covariance function.

The time series analyzed here was characterized by strong autocorrelation. Thus, an autoregressive integrated moving average (ARIMA) model [39] was applied to account for this autocorrelation. An ARIMA model is notated as (*p*, *d*, *q*), where *p* is the autoregressive (AR) order, *d* the differencing order, and *q* the moving average (MA) order. The autocorrelation function and partial autocorrelation function were applied to determine the AR and MA order. Models and coefficients were examined through calculated AIC and the coefficient of determination (R^2^), and the optimal model in each relationship was selected: poultry outbreaks lead human cases; human cases lead poultry outbreaks, wild-bird outbreaks led human cases; human cases lead wild-bird outbreaks; wild-bird outbreaks led poultry outbreaks; and poultry outbreaks lead wild-bird outbreaks. All ARIMA modeling and corresponding statistical tests were performed using R (version 2.13.2).

## Results

### Spatial and temporal pattern of H5N1 outbreak

**Figure 2:**
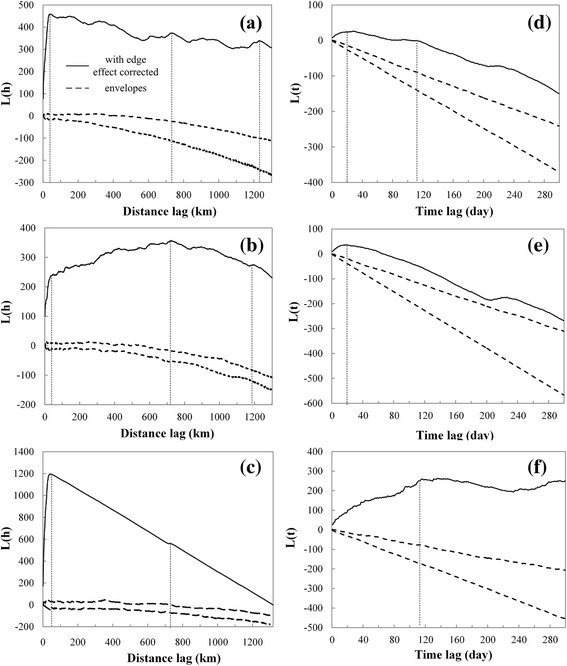
**Temporal and spatial K-function results.** Spatial K-function calculated for: **(a)** all infections, **(b)** poultry and **(c)** wild birds. Temporal K-function calculated for: **(d)** all infections, **(e)** poultry and **(f)** wild birds. Clusters are shown by thin dashed lines. The peak value in spatial K-function for wild birds represents the outbreaks in Hong Kong, because of data bias.

Figure 2a shows values of the spatial K function. There were three peaks located at approximately 50, 700 and 1200 km for the entire dataset. The poultry outbreaks exhibited the same three peaks as the overall trend (Figure 2b), whereas wild-bird outbreaks showed a single peak at 50 km (Figure 2c). The 50-km lag corresponded to the average distance between neighboring counties, within a city or at a specific locale. The 700- and 1200-km lags may fit the distance between provinces. Outbreaks in wild birds were concentrated in the Qinghai-Tibetan Plateau, Hong Kong and Liaoning Province. We suspect that the lag for wild birds was related to their migration distances; the 50-km lag corresponded to an outbreak within a city, or at breeding, wintering and stopover sites.

Figure 2d shows values of the temporal K function. There were two peaks located at approximately 20 and 115 days. Poultry outbreaks exhibited a single peak at 20 days (Figure 2e), while wild-bird outbreaks had a single peak at 115 days (Figure 2f). The 20-day peak may correspond to poultry trade within a province or between provinces. The 115-day peak may fit the migration cycle of migratory birds.

### Correlations between poultry and wild-bird outbreaks and human cases

Outbreak data began in 2004, and there were epidemic waves during four periods in China: January–February 2004, October 2005–June 2006, January–June 2005, and December 2008–February 2009. The outbreaks revealed significant seasonal patterns. Wild-bird outbreaks tended to concentrate in January, February, April and May; poultry outbreaks in January, February, June and November; and human cases in January (Additional file 2: Figure S1).

**Figure 3:**
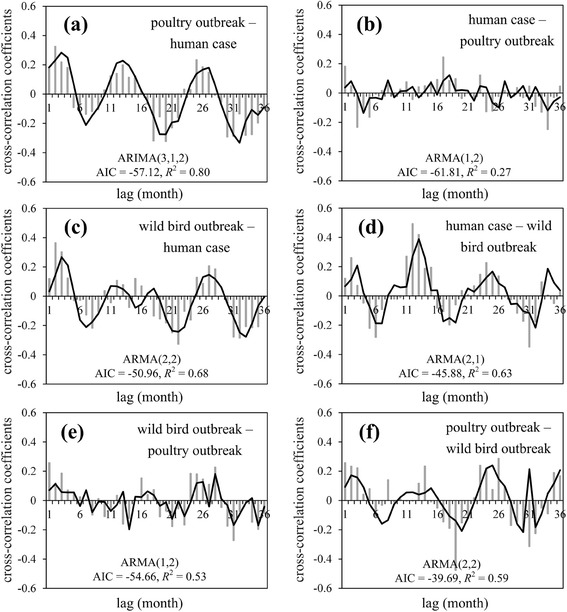
**Cross-correlation coefficient time series and ARIMA model-fitted curves. (a)** Poultry outbreak – human case; **(b)** human case – poultry outbreak; **(c)** wild-bird outbreak – human case; **(d)** human case – wild-bird outbreak; **(e)** wild-bird outbreak – poultry outbreak; and **(f)** poultry outbreak – wild-bird outbreak. Thick lines are best-fit results of cross-correlation coefficient time series by ARIMA modeling.

An ARIMA model was obtained for each of the six relationships (Figure 3), and a total of 384 models (4 × 4 × 4 × 6, *p*: 0–4, *d*: 0–4, *q*:0–4) were tested (Additional file 3: Table S2). The results indicated that human cases were preceded by poultry outbreak with 1–4 months (Figure 3a), and wild-bird outbreaks were led by human cases with 1–3 months (Figure 3d). These lead–lag relationships were considered to be stable, with approximately 12 and 11 months periodicity, respectively.

### Circulation of H5N1 virus in China, 1996–2011

**Figure 4:**
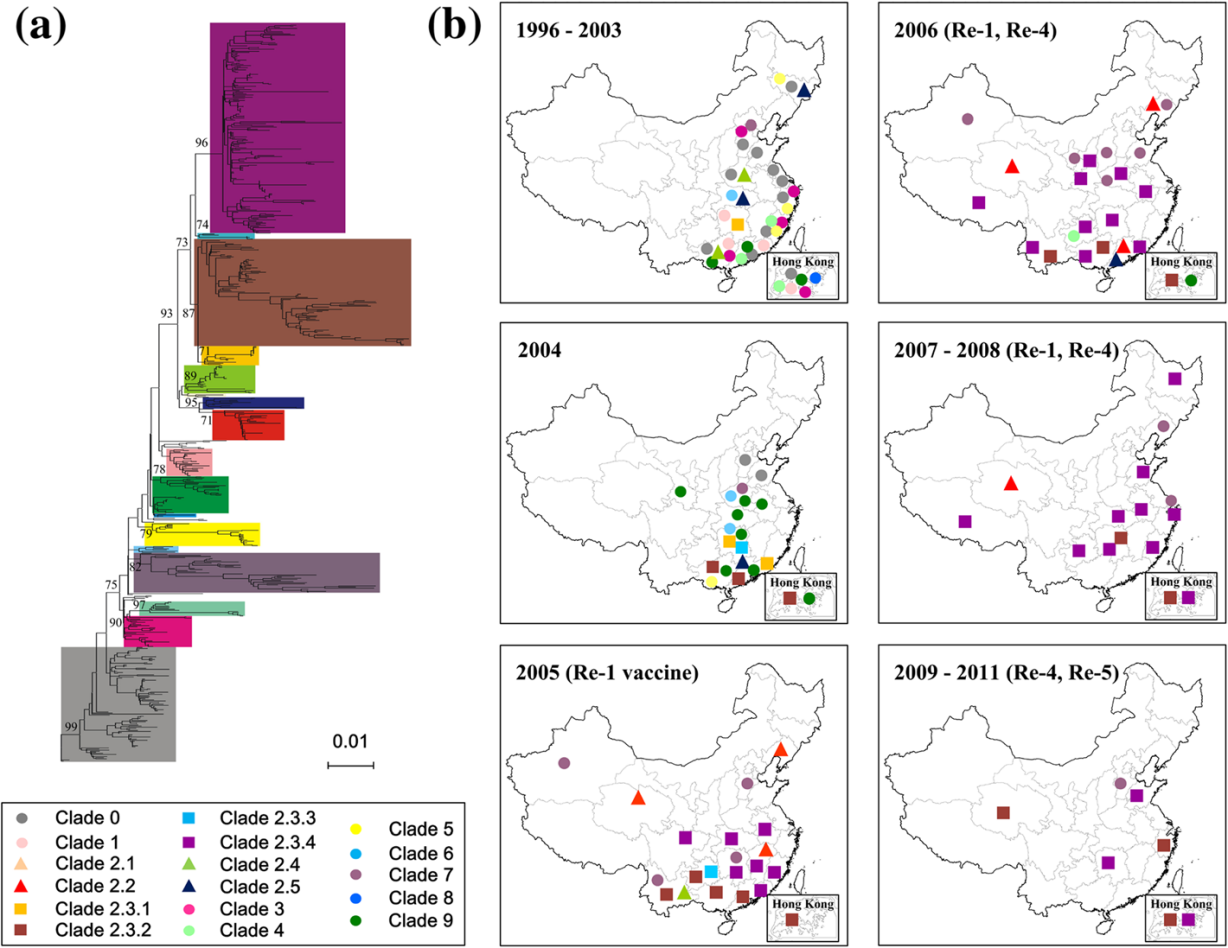
**Spatiotemporal distribution of different virus clades isolated from birds in China, 1996–2011. (a)** Phylogenetic tree based on H5N1 HA gene sequences. **(b)** Distribution of H5N1 virus isolated from birds in China, 1996–2011. The definition criteria of clades were developed by the WHO/OIE/FAO H5N1 Evolution Working Group: sharing of a common (clade-defining) node in the phylogenetic tree; monophyletic grouping with a bootstrap value ≥60 at the clade-defining node; and average percentage pairwise nucleotide distances between and within clades of >1.5% and <1.5%, respectively.

Virus strains in China were classified into different clades according to the supplement from WHO (Figures 4a and Additional file 4: FigureS2). A distribution map of different virus clades based on spatiotemporal information of isolates was drawn (Figure 4b). Clade 0 virus was the circulating strain at the early stage; after 2004, it was replaced and several new clades emerged and began to circulate, such as 2.3.2, 2.3.4, and 7. The virus began to spread westward and has been detected in almost all of China since then.

**Figure 5:**
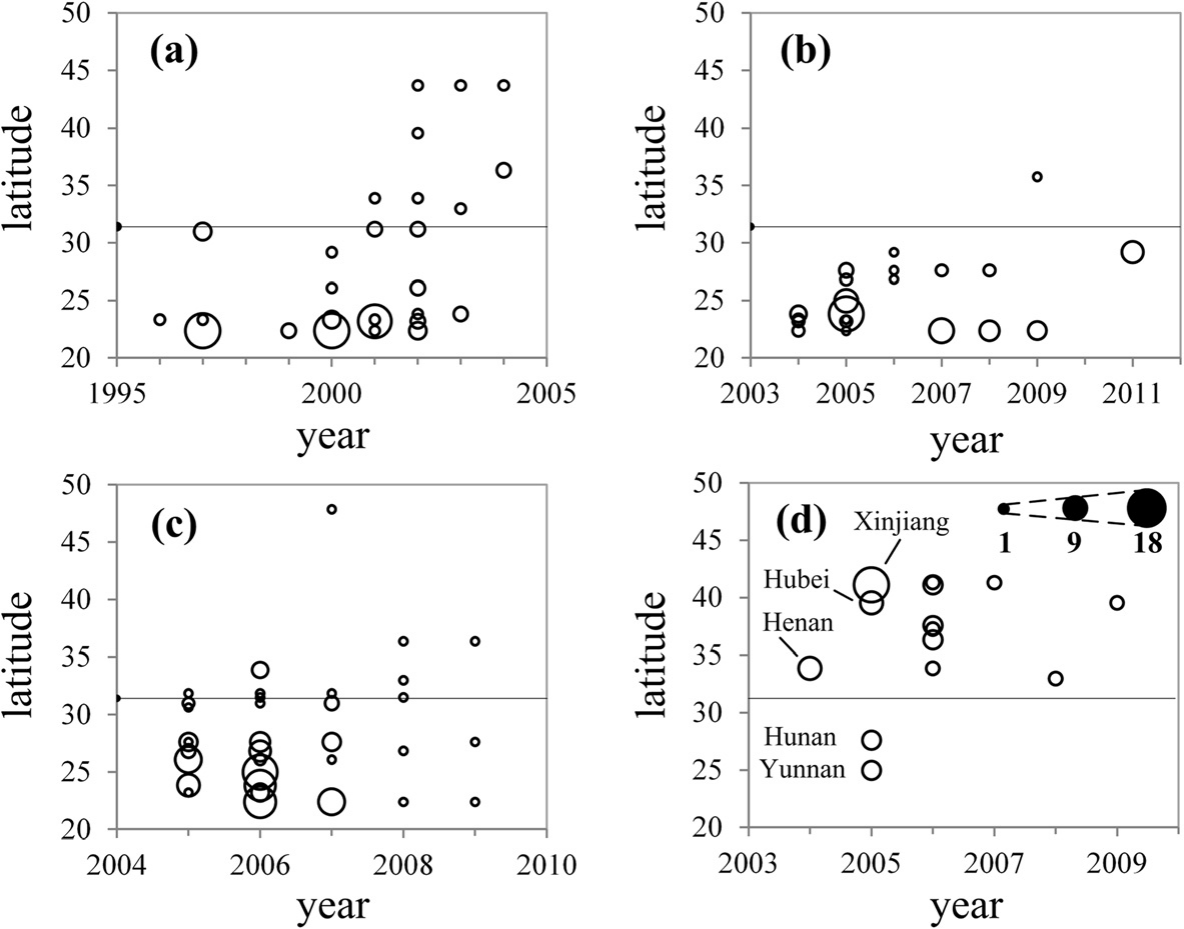
**Distribution of prevailing H5N1 virus clades in China. (a)** Clade 0; **(b)** clade 2.3.2; **(c)** clade 2.3.4; **(d)** clade 7. Thin black line represents latitude of Yangtze River. Circle size is proportional to sample size, with smallest circle representing one sample, and largest 18 samples.

Clade 0 virus circulated in poultry south of the Yangtze River before 2000. The Yangtze River has been historically considered as a geographic middle line dividing the country into north and south. With time, the virus spread north and crossed the Yangtze River in 2001. After 2004, there was no clade 0 virus isolated from birds, indicating that it may have been replaced by other clades (Figure 5a). In 2004, clade 2.3.2 emerged in Southern China and clade 7 in Northern China. These may have taken over the dominant role of clade 0 in those parts of the country.

Clade 2.3.2 virus appeared in 2004 and circulated south of the Yangtze River, except for one wild-bird isolate found in Qinghai in 2009 [40]. After 2006, a variant of this virus, clade 2.3.2.1, began circulating in China and Vietnam, and the regularly-used vaccines did not provide full protection (Figure 5b). Clade 2.3.4 emerged in 2005 and spread from Southern to Northern China. It reached Henan Province, the geographic centroid of the country, in 2006, and appeared in wild birds in Heilongjiang, the northernmost province, in 2007 (Figure 5c).

Clade 7 virus appeared in wild birds in Henan Province in 2004. In the following year the virus spread south, reaching Hunan and Yunnan Provinces; however, it disappeared in these areas in 2006. The virus also spread north, reaching Xinjiang and Hebei Provinces, and became the primary circulating strain in poultry in Northern China (Figure 5d).

After an outbreak in migratory waterfowl at Qinghai Lake in 2005 [41], clade 2.2 virus persisted in the Qinghai–Tibet Plateau region during 2005–2007, and began to decrease in 2008. Surprisingly, clade 2.2 was seldom isolated from poultry in China, and it was not the strain circulating in birds throughout China, although it had a large and long-lasting global impact on various host species.

### Effect of vaccination on evolution of H5N1

Three H5N1 avian influenza vaccines, Re-1, Re-4 and Re-5, were widely adopted in China (Table 1). The Re-1 vaccine was first used at the end of 2004, and for almost 10 years after clade 0 appeared. The Re-4 vaccine was first used in August 2006, 2 years after clade 7 appeared. The Re-5 vaccine was first used in May 2008, 4 years after clade 2.3.4 emerged. After compulsory vaccination began in 2004, several specific sub-clades appeared, such as 2.3.2 and 2.3.4. The mean evolutionary rates of H5N1 virus increased after vaccination was implemented in the country. The mean nucleotide substitution rate for all H5N1 viruses collected from 1996 to 2004 was 3.77 × 10^−3^ substitutions/site/year (95% HPD from 3.33 × 10^−3^ to 4.22 × 10^−3^ substitutions/site/year), and 4.39 × 10^−3^ substitutions/site/year (95% HPD from 4.03 × 10^−3^ to 4.76 × 10^−3^ substitutions/site/year) from 2005 to 2012. The results were consistent with those from other countries where vaccination was adopted [15]. We also conducted a sliding separation window analysis to examine the variation of evolutionary rates over time. The results indicated that evolutionary rates of H5N1 virus increased in the year when the vaccine was used in China (Additional file 5: Figure S3, Table 2).

**Table 1:** **Major vaccine types used for control of H5N1 avian influenza in China from 2004 to 2011**

**Vaccine**	**HA gene donor virus**	**First use**
Re-1	A/goose/Guangdong/1/1996 (Clade 0)	End of 2004
Re-4	A/chicken/Shanxi/2/2006 (Clade 7)	Aug. 2006
Re-5	A/duck/Anhui/1/2006 (Clade 2.3.4)	May 2008

**Table 2:** **Evolutionary profiles of H5N1 HPAI viruses: comparisons between strict and relaxed (uncorrelated lognormal) molecular clocks using the constant, exponential growth, Bayesian skyride and Bayesian skyline coalescent prior**

		**Evolutionary rates (sub/site/year) × 10** ^**−3**^ (95% HPD*)							
	**Best fit clock model**	**Strict clock**				**Uncorrelated lognormal molecular clock (ULCN)**			
		**Constant size**	**Exponential growth**	**Bayesian skyride**	**Bayesian skyline**	**Constant size**	**Exponential growth**	**Bayesian skyride**	**Bayesian skyline**
2000-2001	Strict-Skyride	4.15 (2.97-5.44)	3.49 (2.34-4.76)	3.85 (2.98-4.80)	4.12 (3.07-5.39)	4.36 (2.96-5.98)	4.36 (2.93-5.82)	4.61 (3.25-6.08)	4.65 (2.93-6.37)
2001-2002	Strict-Constant	4.06 (3.03-5.17)	3.24 (2.11-4.48)	3.83 (2.92-4.78)	3.72 (2.73-4.72)	5.17 (3.63-6.74)	4.86 (3.21-6.59)	5.16 (3.58-6.63)	5.48 (3.80-7.18)
2002-2003	Strict-Exponential	2.51 (1.87-3.26)	2.34 (1.68-3.09)	2.29 (1.68-2.93)	2.47 (1.81-3.13)	2.52 (1.78-3.29)	2.97 (2.01-3.94)	2.79 (1.85-3.87)	2.61 (1.77-3.49)
2003-2004	Strict-Constant	4.31 (3.38-5.23)	4.15 (3.31-5.00)	4.19 (3.28-5.12)	4.21 (3.24-5.13)	4.83 (3.76-5.93)	4.84 (3.7-6.22)	1.04 (0.01-5.02)	4.86 (3.38-6.60)
2004-2005	Strict-Skyride	6.81 (5.69-7.95)	6.88 (5.66-8.03)	6.63 (5.73-7.56)	6.70 (5.69-7.67)	8.99 (6.69-11.08)	7.83 (6.31-10)	7.15 (5.55-8.46)	7.37 (4.20-9.98)
2005-2006	Strict-Exponential	3.55 (2.94-4.17)	3.62 (3.09-4.19)	3.85 (2.5-5.14)	3.14 (2.03-3.92)	3.52 (2.73-4.44)	4.31 (3.27-5.38)	4.03 (1.25-5.69)	3.44 (1.29-4.53)
2006-2007	UCLN-Exponential	4.18 (3.28-5.17)	3.76 (2.59-4.68)	4.07 (2.8-5.45)	3.41 (2.28-4.47)	5.72 (3.78-7.44)	5.48 (3.99-6.92)	4.83 (1.25-5.69)	5.61 (2.20-8.40)
2007-2008	Strict-Skyline	4.37 (3.29-5.56)	4.19 (3.06-5.37)	4.52 (3.4-5.62)	4.66 (3.63-5.81)	4.08 (2.76-5.55)	4.58 (3.04-6.27)	4.96 (3.63-6.44)	5.01 (3.22-7.13)
2008-2009	Strict-Exponential	4.91 (3.64-6.12)	4.84 (3.58-6.07)	5.03 (3.85-6.26)	4.74 (3.57-6.02)	5.98 (4.04-8.06)	6.33 (3.94-8.79)	7.31 (4.72-10.08)	6.44 (4.12-8.96)
1996-2004	UCLN-Exponential	3.24 (2.92-3.60)	3.19 (2.86-3.51)	4.03 (3.58-4.52)	3.63 (3.13-4.10)	3.54 (3.08-3.97)	3.77 (3.33-4.22)	4.03 (3.57-4.51)	3.63 (3.13-4.10)
2005-2011	Strict-Constant	4.39 (4.03-4.76)	4.44 (4.06-4.80)	4.35 (3.88-4.81)	4.45 (4.05-4.87)	4.28 (3.67-4.84)	4.26 (3.15-5.11)	4.30 (3.82-4.78)	3.95 (3.58-4.28)

*HPD, highest posterior density.

**Figure 6:**
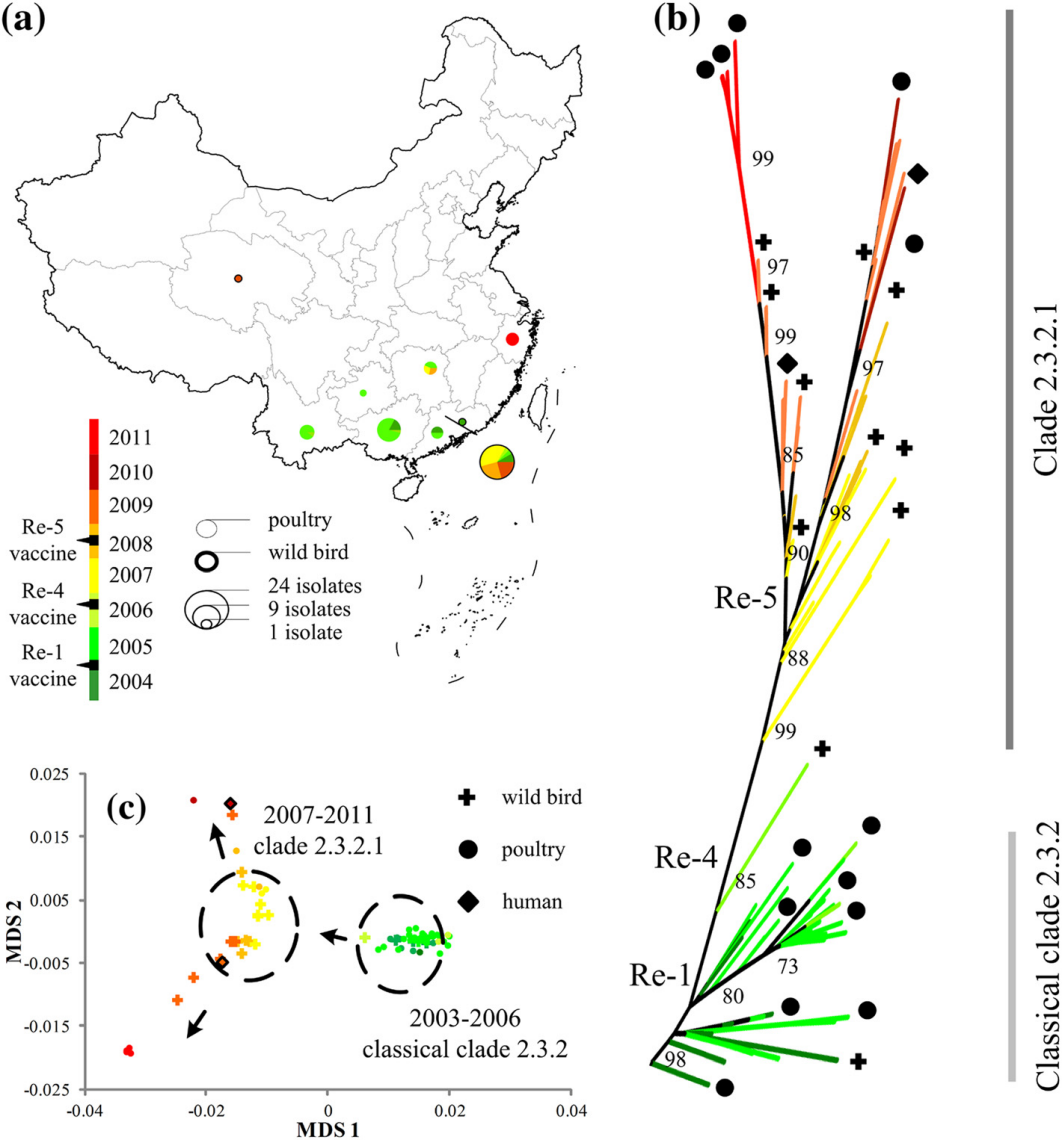
**Clade 2.3.2 virus in China. (a)** Map of China with clade 2.3.2 isolates from animals. **(b)** Phylogenetics of H5N1 HA. **(c)** Genetic map of HA sequences.

To examine further the effect of vaccination on the genomic dynamics of the virus, we focused on clade 2.3.2 that circulated in Southern China, and a total of 97 strains were used for analysis (Additional file 6: Figure S4). The strain appeared in Guangdong and Guangxi Provinces and Hong Kong in 2004. Then it spread to several neighboring provinces of Southern China (Figure 6a). Based on the unrooted tree of clade 2.3.2 in the country, it was clear that the virus was divided into two groups: classical clade 2.3.2 isolated in 2003–2006, and its descendent, clade 2.3.2.1, isolated in 2007–2011. Various host species including poultry, wild birds and humans were infected with these two sub-clades of virus (Figure 6b).

The genetic map (Figure 6c) revealed that there were mainly two distinct clusters with one cluster dominated by poultry stains (81.6% among a total of 49) between 2003 and 2006, and the other cluster dominated by wild-bird strains (40.7% among a total of 27) between 2007 and 2008. The two clusters displayed in the genetic map represented the two groups of sub-clades constructed from the rooted phylogenetic tree. They clearly indicated that the virus might have been transmitted from domestic poultry to wild birds during 2006–2007. Although wild-bird strains have been constantly isolated in Hong Kong and Qinghai between 2007 and 2008, the virus transmission started to return to poultry in 2008 and even the new host of humans in 2009. In 2009–2011, clade 2.3.2.1 developed in two evolutionary directions. The first was in Hunan, Qinghai and Hubei Provinces, whereas the second was in Hong Kong and Zhejiang Province.

**Figure 7:**
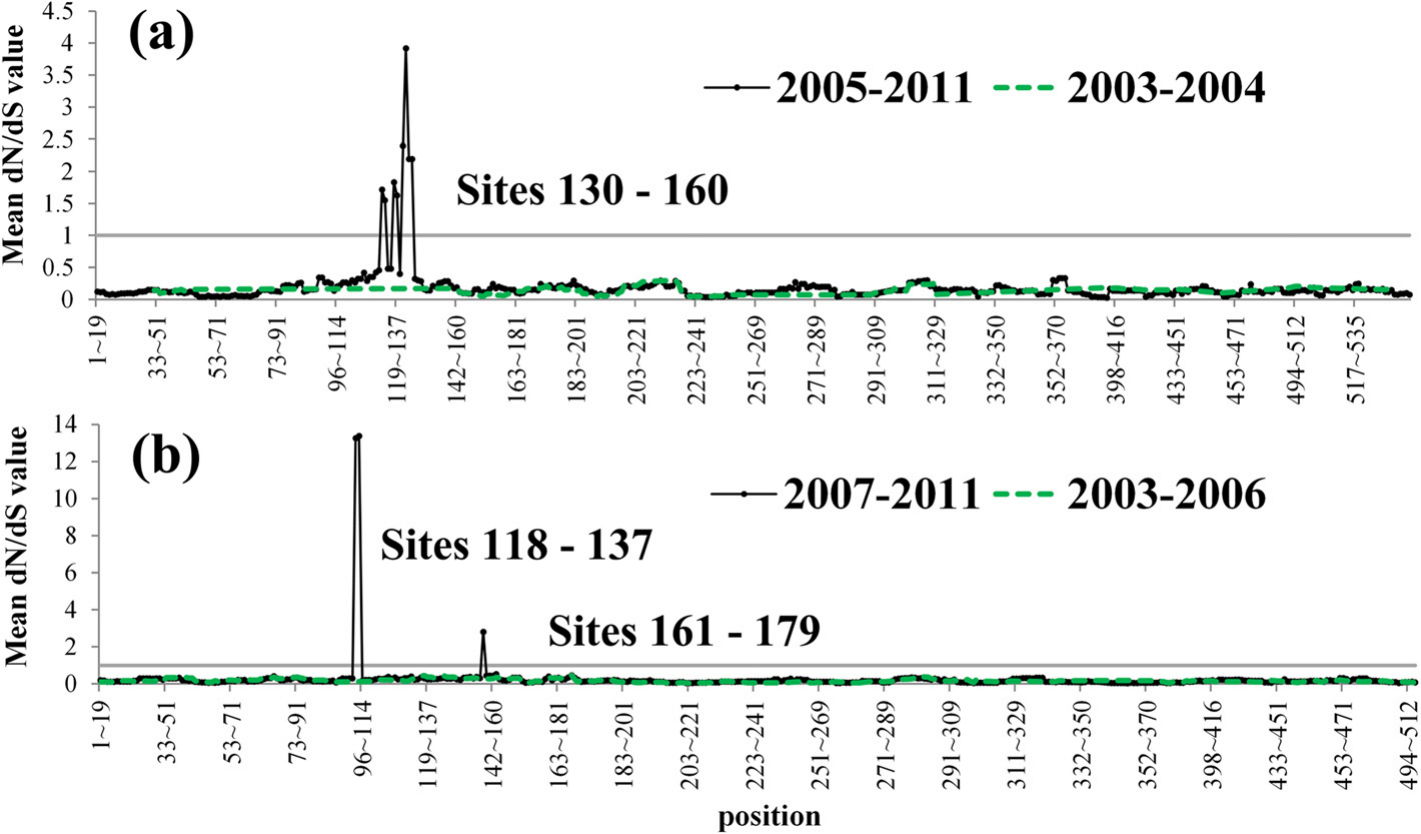
**Detection of positively-selected sites. (a)** Positively Selected Sites. dN/dS ratio compared with Re-1 vaccine. **(b)** dN/dS compared with Re-4 vaccine. Black lines are the mean dN/dS values of virus strains after vaccination was implemented, compared with the corresponding vaccine antigen. Mean dN/dS values of virus before vaccination was implemented are shown by green dashed lines.

The major population divergence of clade 2.3.2 virus was closely related to the beginning of vaccine use in China (Figure 6b, Table 1). The first instance of divergence occurred in 2004, corresponding to the use of Re-1 vaccine; the second in 2006 with Re-4; and the third in 2008 with Re-5 (Figure 6b). The dN/dS ratio comparing clade 2.3.2 virus with the vaccine antigen was calculated. It was found that positions of 130–160 in the HA1 protein were under positive selections during 2005–2011 but not during 2003–2004, suggesting an effect of Re-1 vaccine, which the government started to implement in 2004 (Figure 7a). Similar phenomena occurred again in 2006. Positions 118–137 and 161–179 in the HA1 protein exhibited strong signals of positive selection during 2007–2011 in comparison with those during 2003–2006, suggesting again an effect of Re-4 vaccine, which the government started to implement in 2006 (Figure 7b).

To identify positively-selected sites among codons in the HA1 protein and avoid chance mutations, we divided clade 2.3.2 into three groups based on the timing of three adopted vaccines: unvaccinated group I in 2003–2004; early stage vaccinated group II in 2005–2006 and 2005–2007; and post-vaccination group III in 2007–2008, 2007–2011 and 2009–2011. The results showed no positively-selected sites in group I; the dN/dS ratio over all sites was 0.13–0.19 among models. In group II (2005–2006), model M8 suggested a proportion of sites (2.7%) under positive selection, with dN/dS ratio = 2.56 (Tables 3 and 4). The LRT statistics for comparing M7 and M8 were 2 In*L* = 2 × (In*L*_M8_ – In*L*_M7_) = 6.38 > ${\chi}_{5\%}^2$ = 5.99 with d.f. = 2. For 2005–2007, model M8 suggested a proportion of sites (7.4%) under positive selection, with dN/dS ratio = 1.61 (2 In*L* = 2 × (In*L*_M8_ − In*L*_M7_) = 8.50 > ${\chi}_{5\%}^2$ = 5.99 with d.f. = 2). However, in group III, the model did not detect any positive selection in the dataset. The results indicated that the selected sites occurred after vaccination policy implementation, and the regularly-used vaccines in China did not provide full protection against clade 2.3.2.1 virus after 2007.

**Table 3:** **Positively-selected sites in the HA genes of clade 2.3.2 virus**

**Group**	**Year**	**Vaccine**	**M7**	**M8**	**Positively selected sites by BEB (>50%)** ^**†**^	**Positively selected sites by FEL (** ***P*** **<0.05)** ^**†**^
			**ln** ***L***	**ln** ***L***		
I	2003-2004	None	−2649.7	−2649.6	None	
II	2005-2006	Re-1	−4227.7	−4224.5	145,154,**156**,157,171,**172**,194,204,205	156,171,172,199,205,216
II	2005-2007	Re-1, Re-4	−4872.9	−4868.6	61,136,145,154,156*,157,170,171,172,178,199,200,204,205	56,61,69,131,136,154,156,172,199,204,205
III	2007-2008	Re-1, Re-4	−3807.5	−3806.1	None	
III	2007-2011	Re-1, Re-4, Re-5	−5008.7	−5007.1	None	
III	2009-2011	Re-4, Re-5	−4035.2	−4033.8	None	

Note. Sites inferred under selection at the 90% level are listed in bold, and those at the 95% level are marked with an asterisk.

^†^The posterior probability and *P* value in LRT with null hypothesis of the site are shown in parenthesis for the BEB and FEL analyses, respectively.

**Table 4:** **Parameter estimates for PAML models**

**Group**	**Model**	**Parameter estimates**	Sites* with ω > 1, ω (SE)
II 2005-2006	M7 (β)	*p* = 0.19, *q* = 0.64	
	M8 (β & ω)	*p* = 0.63, *q* = 2.96, *f*_0_ = 0.97	145, 1.379 (0.763)
		ω = 2.56, *f*_1_ = 0.03	154, 1.34 (0.765)
			**156**, 1.748 (0.571)
			157, 1.313 (0.765)
			171, 1.409 (0.714)
			**172**, 1.749 (0.571)
			178, 1.653 (0.636)
			204, 1.429 (0.712)
			205, 1.517 (0.702)
II 2006-2007	M7 (β)	*p* = 0.16, *q* = 0.60	
	M8 (β & ω)	*p* = 1.81, *q* = 13.43, *f*_0_ = 0.93	61, 1.229 (0.634)
		ω = 1.61, *f*_1_ = 0.07	136, 1.506 (0.584)
			145, 1.288 (0.687)
			154, 1.252 (0.688)
			156*, 1.701 (0.454)
			157, 1.211 (0.688)
			170, 1.507 (0.585)
			171, 1.496 (0.588)
			172, 1.632 (0.516)
			178, 1.504 (0.585)
			199, 1.202 (0.63)
			200, 1.358 (0.637)
			204, 1.268 (0.637)
			205, 1.371 (0.636)

*p* and *q* are parameters of the β distribution. *f* is the proportion of sites assigned to an individual ω (= dN/dS) category or to a β distribution with shape parameters *p* and *q*, *f*_1_ = 1 – *f*_0_. Sites inferred under selection at the 90% level are listed in bold, and those at the 95% level are marked with an asterisk.

Some sites in the HA gene (156 and 172 in group II) were consistently detected as positively selected by both the M7 and M8 models in codeml and by the FEL method. We compared the variable sites between clade 2.3.2 (2003–2006) and clade 2.3.2.1 (2007–2011), summarizing the key sites of variation (Table 5). Based on these results (Figure 7a,b, Tables 3 and 5), we believe that the amino acid site 156 was under selective pressure from the vaccine used in China. These results provide strong support for adaptive evolution of the clade 2.3.2 virus, and that it evolved into clade 2.3.2.1 to escape the vaccine pressure since 2006.

**Table 5:** **Key modified sites between the classical clade 2.3.2 and clade 2.3.2.1 virus, China**

**Clade**		**Codon position**							
	**18**	**61**	**69**	**136**	**156**	**178**	**196**	**242**	**279**
Classical 2.3.2	Gln (100%)	Asp (97.96%)	Arg (97.96%)	Ser (97.96%)	Ser (91.84%)	Arg (95.92%)	Asn (53.06%)	Met (95.92%)	Ala (100%)
2.3.2.1	His (100%)	Asn (91.67%)	Lys (100%)	Asp (95.83%)	Asn (93.75%)	Lys (100%)	Asp (95.83%)	Ile (100%)	Thr (91.67%)

Note. Based on the results of Figure 7a and b, we conclude that the virus may have escaped from the vaccine in sites 118–137, 130–160 and 161–179. Table 3 indicates that site 156 received selective pressure with 95% probability. The table summarizes the key sites that varied from the classical clade 2.3.2 virus to clade 2.3.2.1 virus.

## Discussion

In 1996, A/goose/Guangdong/1/1996 (H5N1), the precursor of currently circulating HPAI H5N1 virus was identified in China [42]. The virus has circulated for 17 years, and presented an imminent threat to humans, poultry production, and wild animals in China [43,44]. Our study provides insight into the spatiotemporal pattern of H5N1 outbreaks in China and the dynamics of virus evolution. The results showed obvious spatiotemporal clusters of H5N1 outbreaks on different scales associated with two transmission modes of H5N1 viruses. Viral evolutionary dynamics were analyzed, and the effect of vaccination on virus circulation in China was identified.

Our time series analysis indicated a significant temporal relationship between poultry outbreaks, human cases, and wild-bird outbreaks. The current transmission chain of H7N9 virus in the country coincides with this H5N1 path. Results showed that human cases correlated with poultry outbreaks with a 1–4-month lag, which indicated that the infection may have started in poultry from live-bird markets and then transmitted to humans, even though the timing of wild-bird infection is still uncertain [45,46]. It is postulated that the virus might have undergone a period of time in poultry and the environment without being detected [47,48]. With an increase in imported poultry or active poultry production to meet the demand prior to Lunar New Year activities, most poultry outbreaks occur during these festivals in China [49]. Human infections occurred 1–4 months thereafter because of dense human population, increased exposure, and possibly evolved high-affinity binding of the virus to human receptors. Wild-bird infections might occur last (correlated with human cases with a 1–3-month lag) after exposure and sufficient population of wild species. Many wild bird species winter in Southern China, and start spring migration in early April, and during these periods, wild birds may be infected directly through contact with infected poultry, or the environment [50].

The transmission mode of poultry was faster, with a shorter cycle (Figure 2e). Additionally, the evolving capability for sustained transmission across species barriers represents a major adaptive challenge, because the number of required mutations is often large [51]. However, a vast number of poultry hosts serve as a reservoir, providing a sufficient population basis for accumulating such mutations [52]. Poultry may therefore play a crucial role in the avian influenza epidemics in the country.

Study of the spatiotemporal pattern of outbreaks is important in the prevention and control of epidemics over large regions [53]. The seasonal characteristics of outbreaks in different hosts means that as the season alternates, epidemic areas shift significantly [17]. The epidemic regions will naturally be affected by future climate change [54,55]. Poultry outbreaks concentrate in January, February, June and November. Wild-bird outbreaks concentrate in January and February in Hong Kong, and April and May in Northeastern China and Qinghai–Tibet Plateau. Through spatiotemporal pattern analysis, we can identify possible areas of subsequent outbreaks around epidemic areas. We can calculate an appropriate radius for prevention and culling, and establish early warning systems for regions potentially affected by outbreaks. The results of this study will help to develop appropriate prevention and control policies toward various host outbreaks in different time periods, to avoid the possibility of continuous large outbreaks.

A culling plus vaccination mixed strategy was initiated for control of HPAI H5N1 outbreaks in Mainland China [56]. The actual effect of vaccination in different years and epidemic regions is debated. Although direct association between vaccination and H5N1 virus evolution is difficult to establish, we found that both evolutionary rates and positively selected sites were affected by vaccination for H5N1 in China. OIE has recognized that the emergence of clade 2.3.2.1 virus was one of the genetic mutations occurring as part of neutral virus evolution [57]. However, we believe that the mutation possibly resulted from vaccination, and some mutation sites in clade 2.3.2.1 virus exhibited a strong signature of positive selection, which was against the signature of neutral evolution.

When an outbreak occurs in many regions, simple culling may not be effective. This is also true in some other infectious diseases [12]. In developing countries, controlling virus spread by only long-term, large-scale culling is an immeasurable encumbrance on human living standards [58]. Therefore, vaccination in combination with culling may be the most appropriate way for the country to control infections. The evolutionary patterns and spatiotemporal distribution of the virus are important in making targeted vaccination policy and developing appropriate prevention measures [59]. An appropriate vaccination strategy that includes immunity planning directed at the spatiotemporal distribution of the circulating virus and its possible evolutionary pattern is more important than efficacy of the vaccine itself [60].

The limitations of this study should be mentioned. First, the spatiotemporal information of H5N1 virus depended on sampling. Virus samples may have been concentrated in supposed high-risk areas, which could have led to data bias. Second, H5N1 outbreak information was collected from a passive surveillance system, and data quality varied significantly across provinces.

## Conclusions

In this study, we highlighted two transmission modes on a regional scale through a spatiotemporal analysis, and revealed lead–lag relationships among poultry outbreaks,–human cases and wild-bird outbreaks. We analyzed the circulation of H5N1 virus, and established an effect of vaccination on virus evolution in China. In conclusion, the H5N1 virus has become endemic in China, and variant strains continue to emerge [42,49]. For infection control, we need to develop effective vaccination and culling strategies, based on analysis of outbreaks and evolutionary patterns of H5N1 virus.

## Additional files

Additional file 1: Table S1. Sequences used in this study. Additional file 2: Figure S1. The cobweb chart plot of seasonal distribution of avian influenza A (H5N1). Reported monthly average occurrence of avian influenza A (H5N1) in China, January 2004–December 2011. **(a)** Poultry outbreaks; **(b)** wild-bird outbreaks. Additional file 3: Table S2. Summary of model performance and the estimated coefficients. Additional file 4: Figure S2. Phylogenetic tree based on H5N1 HA gene sequences in China. Additional file 5: Figure S3. Mean rate of nucleotide substitution of all the H5N1 viruses collected from China using the best fit clock model. Additional file 6: Figure S4. Neighbor-joining (NJ) tree of clade 2.3.2 H5N1 HA sequences in China.
