# Supplementary material for: Spatial, temporal and genetic dynamics of highly pathogenic avian influenza A (H5N1) virus in China
Source: BMC Infect Dis. 2015 Feb 13;15:54. doi: 10.1186/s12879-015-0770-x (PMC4329208; doi:10.1186/s12879-015-0770-x)
Supplement: Additional file 1: Table S1. — Sequences used in this study. [file 12879_2015_770_MOESM1_ESM.pdf]

Table S3. Evolutionary profiles of H5N1 HPAI viruses: comparisons between strict and relaxed (uncorrelated lognormal) molecular clocks using the Constant, Exponential growth, Bayesian skyride and Bayesian skyline coalescent prior.

	Best fit clock model	Evolutionary rates (sub/site/year) $\times 10^{-3}$ (95% HPD*)							
		Strict clock				Uncorrelated lognormal molecular clock (ULCN)			
		Constant size	Exponential growth	Bayesian Skyride	Bayesian Skyline	Constant size	Exponential growth	Bayesian Skyride	Bayesian Skyline
2000-2001	Strict-Skyride	4.15(2.97-5.44)	3.49(2.34-4.76)	3.85(2.98-4.80)	4.12(3.07-5.39)	4.36(2.96-5.98)	4.36(2.93-5.82)	4.61(3.25-6.08)	4.65(2.93-6.37)
2001-2002	Strict-Constant	4.06(3.03-5.17)	3.24(2.11-4.48)	3.83(2.92-4.78)	3.72(2.73-4.72)	5.17(3.63-6.74)	4.86(3.21-6.59)	5.16(3.58-6.63)	5.48(3.80-7.18)
2002-2003	Strict-Exponential	2.51(1.87-3.26)	2.34(1.68-3.09)	2.29(1.68-2.93)	2.47(1.81-3.13)	2.52(1.78-3.29)	2.97(2.01-3.94)	2.79(1.85-3.87)	2.61(1.77-3.49)
2003-2004	Strict-Constant	4.31(3.38-5.23)	4.15(3.31-5.00)	4.19(3.28-5.12)	4.21(3.24-5.13)	4.83(3.76-5.93)	4.84(3.7-6.22)	1.04(0.01-5.02)	4.86(3.38-6.60)
2004-2005	Strict-Skyride	6.81(5.69-7.95)	6.88(5.66-8.03)	6.63(5.73-7.56)	6.70(5.69-7.67)	8.99(6.69-11.08)	7.83(6.31-10)	7.15(5.55-8.46)	7.37(4.20-9.98)
2005-2006	Strict-Exponential	3.55(2.94-4.17)	3.62(3.09-4.19)	3.85(2.5-5.14)	3.14(2.03-3.92)	3.52(2.73-4.44)	4.31(3.27-5.38)	4.03(1.25-5.69)	3.44(1.29-4.53)
2006-2007	UCLN-Exponential	4.18(3.28-5.17)	3.76(2.59-4.68)	4.07(2.8-5.45)	3.41(2.28-4.47)	5.72(3.78-7.44)	5.48(3.99-6.92)	4.83(1.25-5.69)	5.61(2.20-8.40)
2007-2008	Strict-Skyline	4.37(3.29-5.56)	4.19(3.06-5.37)	4.52(3.4-5.62)	4.66(3.63-5.81)	4.08(2.76-5.55)	4.58(3.04-6.27)	4.96(3.63-6.44)	5.01(3.22-7.13)
2008-2009	Strict-Exponential	4.91(3.64-6.12)	4.84(3.58-6.07)	5.03(3.85-6.26)	4.74(3.57-6.02)	5.98(4.04-8.06)	6.33(3.94-8.79)	7.31(4.72-10.08)	6.44(4.12-8.96)
1996-2004	UCLN-Exponential	3.24(2.92-3.60)	3.19(2.86-3.51)	4.03(3.58-4.52)	3.63(3.13-4.10)	3.54(3.08-3.97)	3.77(3.33-4.22)	4.03(3.57-4.51)	3.63(3.13-4.10)
2005-2011	Strict-Constant	4.39(4.03-4.76)	4.44(4.06-4.80)	4.35(3.88-4.81)	4.45(4.05-4.87)	4.28(3.67-4.84)	4.26(3.15-5.11)	4.30(3.82-4.78)	3.95(3.58-4.28)

*HPD, highest posterior density.
