# Supplementary figures and images for: Spatial, temporal and genetic dynamics of highly pathogenic avian influenza A (H5N1) virus in China
Source: BMC Infect Dis. 2015 Feb 13;15:54. doi: 10.1186/s12879-015-0770-x (PMC4329208; doi:10.1186/s12879-015-0770-x)

**(a)**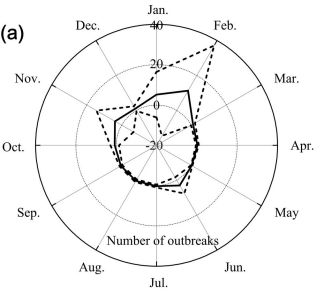**(b)**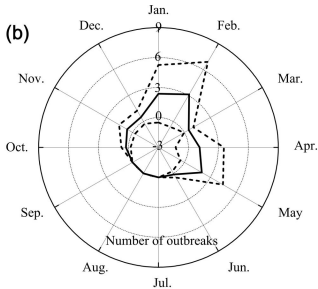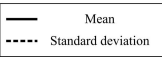

Supplement: Additional file 2: Figure S1. — The cobweb chart plot of seasonal distribution of avian influenza A (H5N1). Reported monthly average occurrence of avian influenza A (H5N1) in China, January 2004–December 2011. (a) Poultry outbreaks; (b) wild-bird outbreaks. [file 12879_2015_770_MOESM2_ESM.pdf]

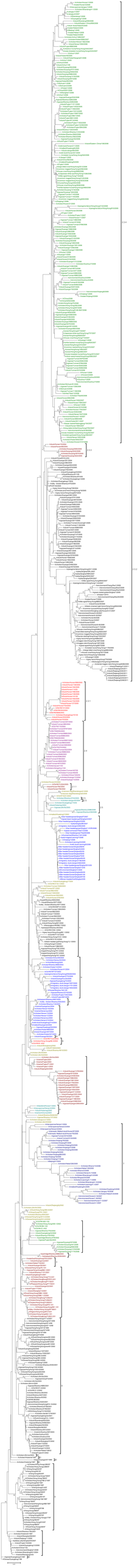

Supplement: Additional file 4: Figure S2. — Phylogenetic tree based on H5N1 HA gene sequences in China. [file 12879_2015_770_MOESM4_ESM.pdf]

Evolutionary rate

sub/site/year ( $\times 10^{-3}$ )

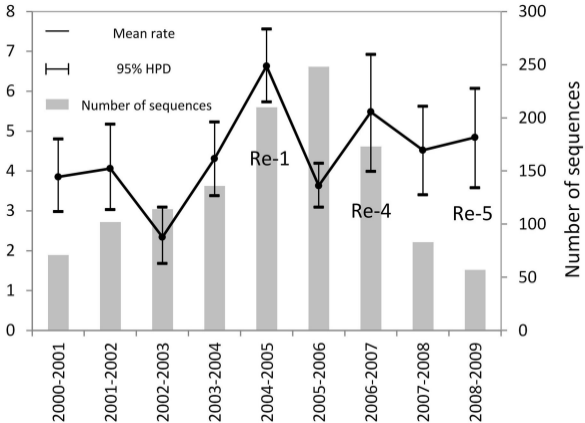

Supplement: Additional file 5: Figure S3. — Mean rate of nucleotide substitution of all the H5N1 viruses collected from China using the best fit clock model. [file 12879_2015_770_MOESM5_ESM.pdf]

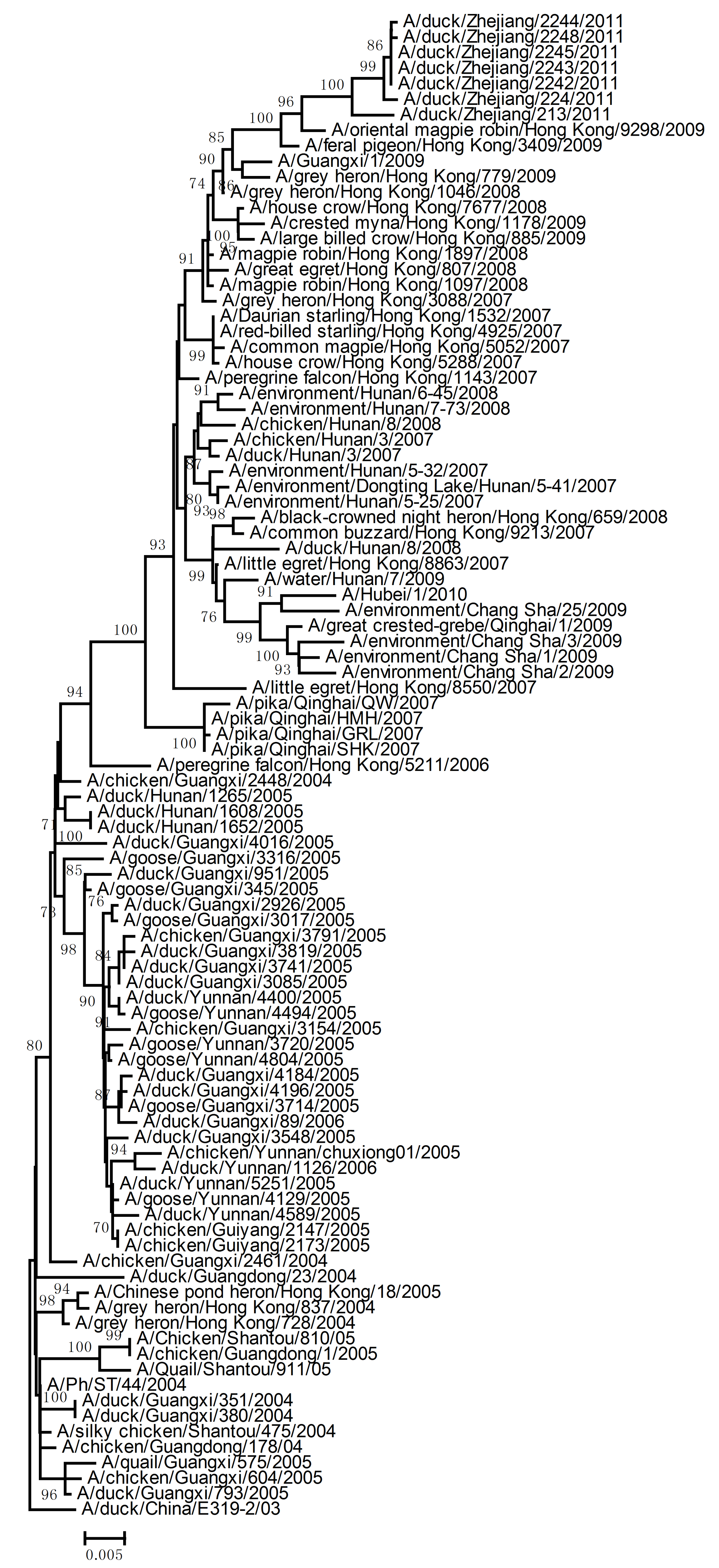

Supplement: Additional file 6: Figure S4. — Neighbor-joining (NJ) tree of clade 2.3.2 H5N1 HA sequences in China. [file 12879_2015_770_MOESM6_ESM.tiff]
