# Supplementary material for: Spatial, temporal and genetic dynamics of highly pathogenic avian influenza A (H5N1) virus in China
Source: BMC Infect Dis. 2015 Feb 13;15:54. doi: 10.1186/s12879-015-0770-x (PMC4329208; doi:10.1186/s12879-015-0770-x)
Supplement: Additional file 3: Table S2. — Summary of model performance and the estimated coefficients. [file 12879_2015_770_MOESM3_ESM.pdf]

Table S3. Parameter estimates for PAML models.

Group	Model	Parameter estimates	Sites* with ω > 1, ω (SE)
II 2005-2006	M7 (β)	p = 0.19, q = 0.64	
	M8 (β & ω)	p = 0.63, q = 2.96, f_0 = 0.97	145, 1.379 (0.763)
		ω = 2.56, f_1 = 0.03	154, 1.34 (0.765)
			**156**, 1.748 (0.571)
			157, 1.313 (0.765)
			171, 1.409 (0.714)
			**172**, 1.749 (0.571)
			178, 1.653 (0.636)
			204, 1.429 (0.712)
			205, 1.517 (0.702)
II 2006-2007	M7 (β)	p = 0.16, q = 0.60	
	M8 (β & ω)	p = 1.81, q = 13.43, f_0 = 0.93	61, 1.229 (0.634)
		ω = 1.61, f_1 = 0.07	136, 1.506 (0.584)
			145, 1.288 (0.687)
			154, 1.252 (0.688)
			**156***, 1.701 (0.454)
			157, 1.211 (0.688)
			170, 1.507 (0.585)
			171, 1.496 (0.588)
			172, 1.632 (0.516)
			178, 1.504 (0.585)
			199, 1.202 (0.63)
			200, 1.358 (0.637)
			204, 1.268 (0.637)
			205, 1.371 (0.636)

p and q are parameters of the β distribution. f is the proportion of sites assigned to an individual ω (= dN/dS) category or to a β distribution with shape parameters p and q, $f_1 = 1 - f_0$. Sites inferred under selection at the 90% level are listed in bold, and those at the 95% level are marked with an asterisk.
